# Hyperoside Downregulates the Receptor for Advanced Glycation End Products (RAGE) and Promotes Proliferation in ECV304 Cells via the c-Jun *N*-Terminal Kinases (JNK) Pathway Following Stimulation by Advanced Glycation End-Products *In Vitro*

**DOI:** 10.3390/ijms141122697

**Published:** 2013-11-18

**Authors:** Zhengyu Zhang, Mosha Silas Sethiel, Weizhi Shen, Sentai Liao, Yuxiao Zou

**Affiliations:** 1 Sericulture & Agri-Food Research Institute, Guangdong Academy of Agricultural Sciences, NO. 133 Yiheng St. Dongguanzhuang Rd., Tianhe Ditrict, Guangzhou 510610, China; E-Mails: skyforce12@163.com (W.S.); liaost@163.com (S.L.); 2 Department of Histology and Embryology, Guangzhou Medical University, Guangzhou 510185, China; E-Mails: zhengyuceo@163.com (Z.Z.); snclrmosha@gmail.com (M.S.S.)

**Keywords:** hyperoside, RAGE, AGE, JNK, ECV304

## Abstract

Hyperoside is a major active constituent in many medicinal plants which are traditionally used in Chinese medicines for their neuroprotective, anti-inflammatory and antioxidative effects. The molecular mechanisms underlying these effects are unknown. In this study, quiescent ECV304 cells were treated *in vitro* with advanced glycation end products (AGEs) in the presence or absence of hyperoside. The results demonstrated that AGEs induced c-Jun *N*-terminal kinases (JNK) activation and apoptosis in ECV304 cells. Hyperoside inhibited these effects and promoted ECV304 cell proliferation. Furthermore, hyperoside significantly inhibited RAGE expression in AGE-stimulated ECV304 cells, whereas knockdown of RAGE inhibited AGE-induced JNK activation. These results suggested that AGEs may promote JNK activation, leading to viability inhibition of ECV304 cells via the RAGE signaling pathway. These effects could be inhibited by hyperoside. Our findings suggest a novel role for hyperoside in the treatment and prevention of diabetes.

## 1. Introduction

Hyperoside is a flavonoid compound mainly found in herbal plants which are traditionally used in Chinese medicines for their neuroprotective, anti-inflammatory, antioxidative and vascular protective effects. However, the molecular mechanism underlying these actions is unknown.

Endothelial dysfunction is a key triggering event in atherosclerosis. Treatment with hyperoside has been found to attenuate endothelial cell damage induced by oxidative stress [1]. Some studies have shown that hyperoside significantly decreases total cholesterol, triglyceride, low density lipoprotein and very low density lipoprotein levels in serum, coupled with elevation of high density lipoprotein in diabetic rats [2].

The binding of advanced glycation end products (AGE) to the receptor for AGE (RAGE) is known to deteriorate various cell functions and is implicated in the pathogenesis of diabetic vascular complications [3]. AGEs activate the RAGE gene through NF-kappaB (NF-κB) and Sp-1 in ECV304 cells, thereby enhancing AGE-RAGE interactions, which can lead to an exacerbation of diabetic microvasculopathy [4]. It is unclear whether hyperoside can inhibit apoptosis in ECV304 cells in response to AGEs.

We hypothesized that RAGE could mediate the intracellular signals in ECV304 cells initiated by AGEs, resulting in activation of the c-Jun *N*-terminal kinases (JNK) pathway and apoptosis in ECV304 cells. Our findings indicated that hyperoside may inhibit JNK activation and promote ECV304 cell proliferation by downregulating RAGE.

## 2. Results and Discussion

### 2.1. Hyperoside Promotes Cell Viability and Attenuates the Effects of AGEs (MTT)

**Figure 1:**
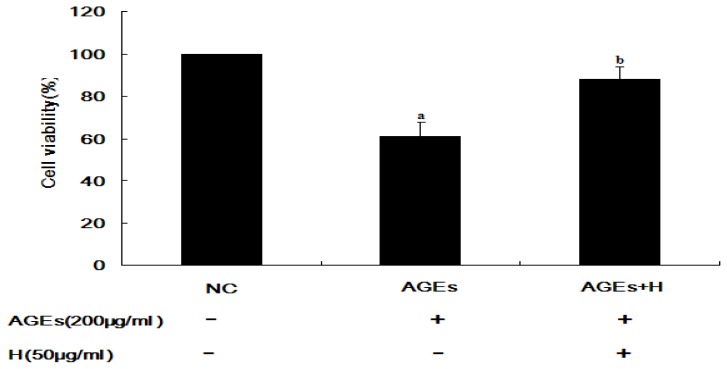
Hyperoside promotes cell viability and attenuates the effects of advanced glycation end products (AGEs). Cells were seeded in 96-well culture plates. After incubation at 37 °C for 24 h, the medium was replaced with fresh medium supplemented either with or without hyperoside (50 μg/mL). After incubation for a further hour, AGEs (200 μg/mL) were added to the cells for 10 min. The viability of ECV304 cells were detected after 24 h treatment with AGEs, either in the absence or presence of hyperoside. The results are shown as a percentage of negative control. (**a**) AGEs *vs.* NC (*p* < 0.01); (**b**) AGEs + H *vs.* AGEs (*p* < 0.05). NC, negative control; H, hyperoside.

It has been reported that AGE inhibits proliferation of ECV304 cells [5,6]. However, it is unknown whether hyperoside can inhibit these effects. A significant difference in cell viability between the AGE-treated and negative control (NC) groups was observed (*p* < 0.01), indicating that treatment with AGEs could inhibit cell viability in ECV304 cells. In contrast, there was a significant difference between cells treated with AGEs and hyperoside compared to those treated with AGEs alone (*p* < 0.05; Figure 1), indicating that hyperoside promoted cell viability.

### 2.2. Hyperoside Inhibits AGE-Induced Activation of JNK in ECV304 Cells

**Figure 2:**
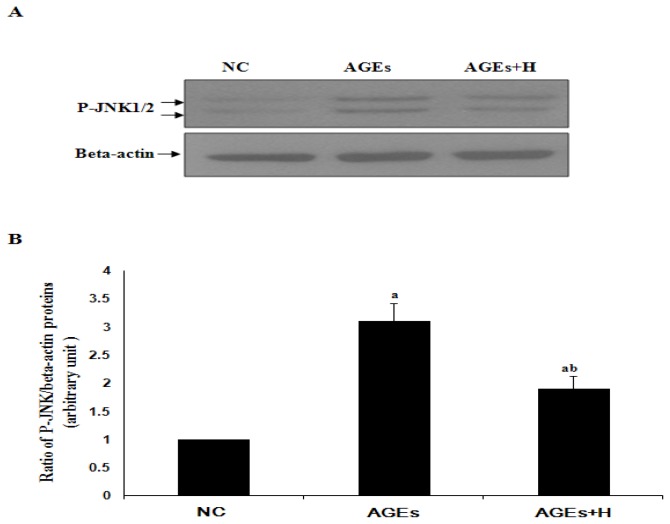
Hyperoside inhibits AGE-induced activation of JNK in ECV304 cells. (**A**) Western blotting shows the activation of c-Jun *N*-terminal kinases (JNK) (P-JNK) in ECV304 cells in response to AGEs in the absence or presence of hyperoside; (**B**) The statistical results of (A) show (**a**) *vs.* NC (*p* < 0.05); and (**b**) AGEs + H *vs.* AGEs (*p* < 0.05). Results are from three independent experiments; β-actin was used as an internal control. NC, negative control; H, hyperoside.

To date, there are no data available on the effects of hyperoside in AGE-induced activation of JNK in ECV304 cells. P-JNK is the activated JNK. When JNK was activated by some kinds of stimulations, more P-JNK can be detected. As shown by Western blot analysis (Figure 2), the increase in JNK activation induced by AGEs was significantly inhibited by hyperoside in ECV304 cells (*p* < 0.05). This suggests that hyperoside may have an inhibitory effect on JNK activation.

### 2.3. Hyperoside Downregulates AGE-Induced Expression of RAGE in ECV304 Cells

**Figure 3:**
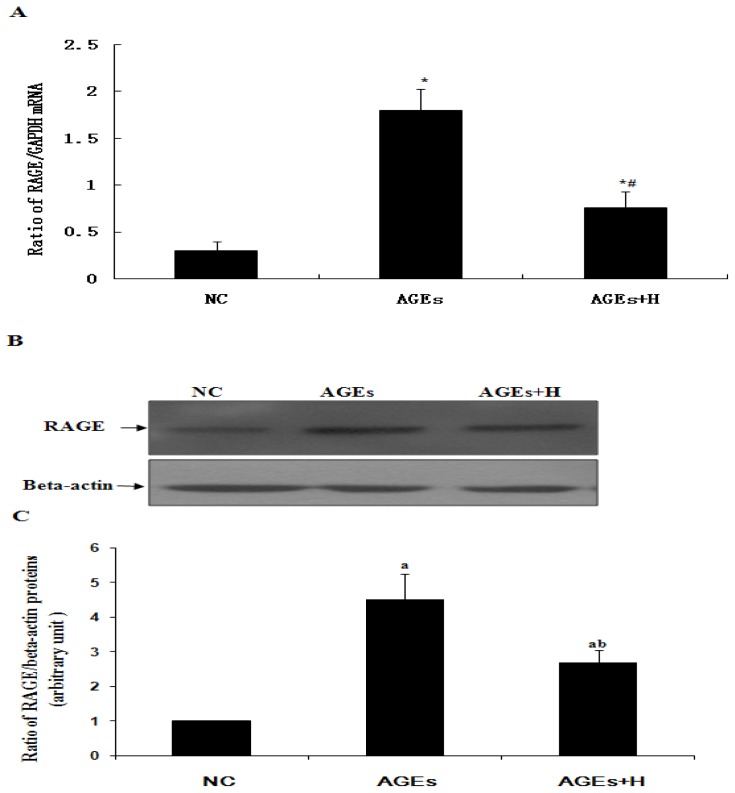
Hyperoside downregulates expression of RAGE induced by AGEs in ECV304 cell. (**A**) qPCR shows the expression levels of RAGE in ECV304 cells treated by AGEs in the absence or presence of hyperoside. (*) *vs.* NC (*p* < 0.05); and (^#^) AGEs + H *vs.* AGEs (*p* < 0.05); (**B**) Western blotting shows the expression levels of RAGE in ECV304 cells treated by AGEs in the absence or presence of hyperoside; (**C**) The statistical results of (**B**) show (**a**) *vs.* NC (*p* < 0.05); and (**b**) AGEs + H *vs.* AGEs (*p* < 0.05). Results are from three independent experiments; β-actin was used as an internal control. NC, negative control; H, hyperoside.

In order to determine whether hyperoside affected RAGE expression induced by AGEs, quiescent ECV304 cells were treated with AGEs either with or without hyperoside. As shown by real time qPCR and Western blot analysis (Figure 3), in the absence of hyperoside AGEs significantly increased expression of RAGE relative to negative control (*p* < 0.05). In contrast, this effect was significantly inhibited by hyperoside (*p* < 0.05). These results suggest that AGEs may induce upregulation of RAGE and that this effect can be inhibited by hyperoside.

### 2.4. Knockdown of RAGE Inhibits AGE-Induced Activation of JNK in ECV304 Cells

**Figure 4:**
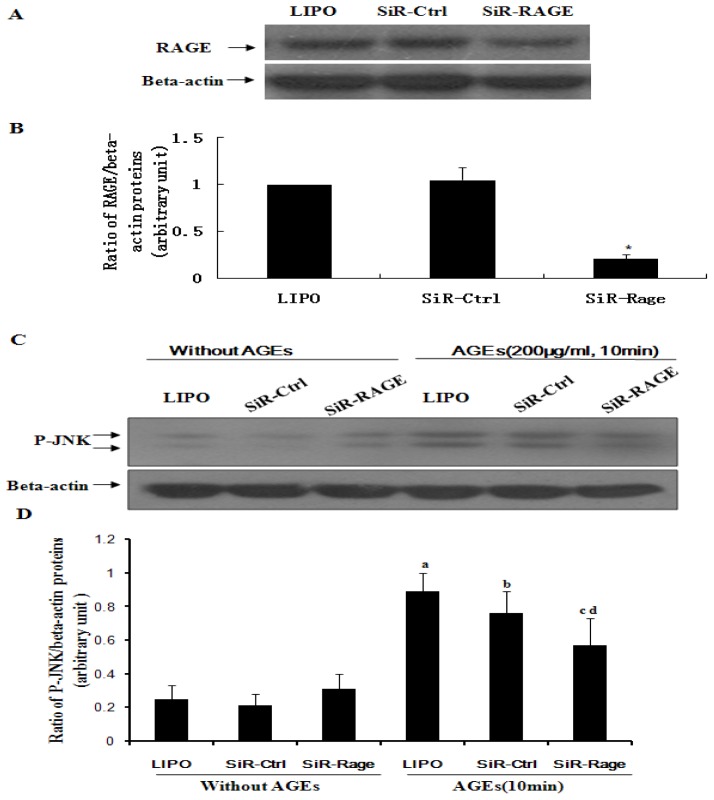
Knockdown of the receptor for AGE (RAGE) inhibits AGE-induced activation of JNK in ECV304 cells. (**A**) Western blotting shows the effect of siRNA-RAGE on expression of RAGE; (**C**) Western blotting shows that knockdown of RAGE via siRNA-RAGE results in significant inhibition of JNK activation induced by AGEs; (**B**,**D**) The statistical results of (A) and (C) show (*) siRNA-RAGE (SIR-Rage) *vs.* siRNA control (Sir-Ctrl); AGEs *vs.* (**a**) Lipofectamine 2000 control (Lipo); (**b**) Non-targeting siRNA control (Sir-Ctrl); and (**c**,**d**) siRNA-RAGE (SIR-Rage) or Sir-Ctrl. All results gave *p* < 0.05. Results are from three independent experiments; β-actin was used as an internal control.

To further assess the contribution of RAGE to AGE-induced activation of JNK, quiescent ECV304 cells transfected with siRNA-RAGE were treated with AGEs either with or without hyperoside. As shown by Western blot analysis (Figure 4), activation of JNK in ECV304 cells was significantly inhibited by knockdown of RAGE relative to a control group (*p* < 0.05). These results suggest that RAGE may mediate JNK signaling induced by AGEs.

### 2.5. Discussion

Human cell line ECV304, derived from urinary bladder carcinoma, showed some biomarkers expected of endothelial cell lines, namely Factor VIII, tubule formation on Matrigel, and/or Weibel-Palade bodies, and so ECV304 cells were selected to study the functions of endothelium cells.

RAGE belongs to the immunoglobulin superfamily of cell surface molecules [7]. It is expressed in multiple tissues [8] and interacts with various ligands including AGE [9,10]. The engagement of RAGE by AGEs has been reported to induce cellular oxidant stress via activation of the transcription factor nuclear factor-κB (NF-κB) [11,12], which evokes inflammatory and thrombogenic responses in various types of cells, including endothelial cells, smooth muscle cells, macrophages and renal cells, leading to the perturbation of a variety of vascular homeostatic functions. AGEs impair cell functions, such as proliferation, migration, apoptosis and adhesion. Abnormal proliferation and migration of these cells directly increase the thickness of the intima and accelerate the formation of atherosclerotic plaque. On the contrary, abnormal apoptosis of these cells leads to rupture of plaque. As such, the AGE-RAGE interaction is thought to play a central role in the development and progression of diabetic vasculopathy [4].

RAGE can engage with AGEs, thereby promoting activation of JNK (Figure 2). It is widely known that activation of JNK is an important step in the induction of cell apoptosis. Finally, a decrease in cell viability was observed (Figure 1). At the same time, in agreement with Tanaka *et al.* [4] and Shi L. *et al.* [13], our results confirmed that AGEs can induce upregulation of RAGE in ECV304 cells (Figure 3), which maybe promote cell apoptosis by the AGEs/RAGE/JNK pathway, because there is more RAGE that can engage with AGEs. In contrast, knockdown of RAGE expression with siRNA-RAGE significantly inhibited AGE-induced JNK activation in ECV304 cells (Figure 4). These results suggested that AGEs may play an important role in the genesis and progression of atherosclerosis via the RAGE/JNK signaling pathway.

The expression of vascular RAGE can be inhibited by clinical drugs and compounds. These include antihypertensive drugs (Telmisartan [14] and Candesartan [15]), vitamin A [16], selenium [17], n-3 polyunsaturated fatty acids [18], antihyperlipidemic agents (statins) [19], antidiabetic agents (thiazolidinediones) [20], peroxisome proliferator-activated receptor gamma [21] and several extracts from Chinese traditional medicines, such as ginkgo biloba extract [22] and Panax notoginseng saponins (PNS) [23]. This suggests that reduced expression of RAGE may contribute to the therapeutic value of these drugs.

Hyperoside is widely used in clinical practice to relieve pain and improve cardiovascular functions. It can protect cortical neurons from oxygen-glucose deprivation-reperfusion induced injury via the nitric oxide signaling pathway and the extracellular signal-regulated kinases (ERK), JNK and Bcl-2 family-related apoptotic signaling pathways [24]. In this study, hyperoside was found to suppress RAGE expression, resulting in a significant decrease in AGE-induced activation of JNK to promote proliferation in ECV304 cells (Figures 1–3). These results outline a new mechanism of the pleiotropic effects of hyperoside in ECV304 cell proliferation in response to AGEs.

## 3. Materials and Methods

### 3.1. ECV304 Cell Culture

Human cell line ECV304, derived from urinary bladder carcinoma was purchased from the Shanghai Institute of Cell Biology (SIBCB; Shanghai Institutes for Biological Sciences, Chinese Academy of Sciences, Shanghai, China). The cells were cultured in RPMI 1640 (Life Technologies, Carlsbad, CA, USA) supplemented with 10% fetal calf serum, penicillin and streptomycin at 37 °C in a humidified atmosphere of 5% CO_2_. The medium was changed every 2 days and the cells were passage by treatment with 0.05% trypsin-0.02% EDTA. Experiments were performed on ECV304 cells at the point of confluence.

### 3.2. AGE Preparation

AGEs were prepared in a manner similar to that described by Kim *et al.* [25]. Briefly, 1 mM fatty acid-free bovine serum albumin (BSA) was dissolved in phosphate buffered saline (PBS) with 0.5 M glucose and incubated under sterile condition for 8 weeks at 37 °C. Reaction mixtures were dialyzed against PBS to remove free glucose and then passed through an affinity column (Pierce, Rockford, IL, USA) to remove any endotoxins. Non-glycated BSA was subjected to the same conditions with the exception of glucose as a control. AGEs were identified by fluorescence spectrophotometry [26].

### 3.3. RNA Interference

The procedures used in this experiment were similar to those described in our previous report [27]. The following RAGE small interfering RNA (siRNA-RAGE) was synthesized by Invitrogen (Carlsbad, CA, USA): sense, 5′-GACCAACUCUCUCCUGUAUTT-3′; antisense, 5′-AUACAGGAGAGAGUUGGUCTT-3′. A non-targeting siRNA duplex sequence (Invitrogen Stealth RNAi, Carlsbad, CA, USA) was used as a negative control. ECV304 cell transfection was performed according to the manufacturer’s instructions. Serum-starved ECV304 cells were subjected to AGEs and/or hyperoside for 10 min for Western blot analysis, or cultured for an additional 24 h for MTT assays.

### 3.4. Cell Viability Assay (MTT)

Cell viability was determined using a CellTiter 96 Non-Radioactive Cell Proliferation Assay kit (Promega, Madison, WI, USA) according to the manufacturer’s instructions. Briefly, EVC304 cells were seeded in 96-well culture plates at 3000 cells/well. After incubation at 37 °C for 24 h, the medium was replaced with fresh medium supplemented either with or without hyperoside (50 μg/mL). After incubation for a further hour, AGEs (200 μg/mL) were added to the cells for 10 min. Dye solution was added to each well 24 h after treatment and the cells were incubated at 37 °C for 2 h. Solubilization/Stop Solution was added to each well and the absorbance was measured at 570 nm using an ELISA reader. Relative cell numbers were calculated after normalizing the absorbance to untreated cells. Cell viability was calculated relative to untreated cells.

### 3.5. Knockdown of RAGE in ECV304 Cells

An experimental system to study of the effects of RAGE on JNK activation in response to AGEs was established by transfecting quiescent ECV304 cells with siRNA-RAGE (100 pmol/well). The cells transfected were then treated with AGEs and/or hyperoside for 10 min. Lipofectamine 2000 and non-targeting siRNA were used as controls

### 3.6. Western Blot Analysis

Western blot analysis was performed as previously described [28], with minor modifications. Treated ECV304 cells were harvested in lysis buffer using protease inhibitors. The lysate suspension was centrifuged and protein concentration was assessed by Bio-Rad protein assay. Heat-denatured proteins were resolved by SDS-PAGE and electrophoretically transferred to nitrocellulose membranes. The membranes were probed with antibodies against phosphorylated JNK (pJNK; Cell Signaling Technology, Danvers, MA, USA) and RAGE (R & D systems, Minneapolis, MN, USA) and reprobed with β-actin antibody (Cell Signaling Technology, Danvers, MA, USA). The bands were visualized using an enhanced chemiluminescence (ECL) detection system (GE Healthcare, Piscataway, NJ, USA). The intensities of the bands were quantified by densitometry.

### 3.7. Real-Time qPCR

qPCR was performed similar to those described previously[29]. In brief, it was performed with a Power SYBR Green PCR Master Mix (Takara, Dalian, China) on an ABI 7900 Real-time PCR instrument according to the manufacturer’s instructions (Applied Biosystems, Foster City, CA, USA). The primer sequences used in this study were designed using Primer 3 (Whitehead Institute/MIT, Cambridge, MA, USA) and confirmed by BLAST in GenBank. Primers of qPCR for amplification of cDNA for RAGE and glyceraldehyde-3-phosphate dehydrogenase (GAPDH) were synthesized by TaKaRa Biotechnology (Dalian, China) as follows: for GAPDH (228 bp) F-5′-CGACCACTTTGTCAAGC TCA-3′, R-5′-AGGGGTCTACATGGCAACTG-3′, for RAGE (143bp) F-5′-AGGAGCGTGCAG AACTGA AT-3′, R-5′-GAGTTGGTCTGAGGCCAGAA-3′.

### 3.8. Statistical Analysis

Data from three independent experiments are expressed as mean ± SD. Statistical analyses were performed by one-way analysis of variance (ANOVA). *p*-values <0.05 were considered significant.

## 4. Conclusions

**Figure 5:**
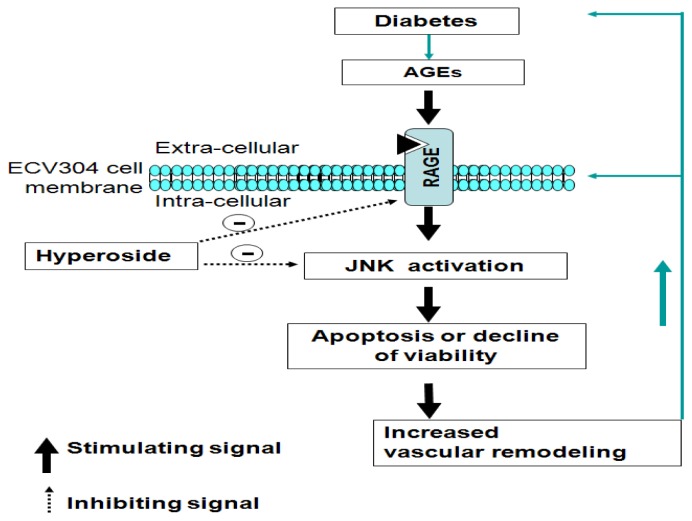
Diagram showing the potential role of the RAGE signaling pathway in the actions of AGEs on JNK activation and ECV304 viability inhibition. Increased blood sugar (diabetes) can trigger rapid increases in AGEs on the walls of vein grafts and arteries. As the ligand of RAGE, AGEs can cause deformation of ECV304 cells, activating RAGE and its downstream signaling molecules, including JNK. Activated JNK (P-JNK) leads to overexpression of RAGE and viability inhibition of ECV304 cells (MTT), thereby altering vascular structure and function. Blocking RAGE and its downstream molecules by hyperoside might inhibit vascular remodeling induced by AGEs.

This study has demonstrated a potential new mechanism for hyperoside in mediating the intracellular signaling pathways initiated by AGEs by inhibiting the effects of RAGE (Figure 5), thereby suppressing JNK activation and increasing cell proliferation in ECV304 cells. This suggests a novel role for hyperoside through blockade of the RAGE downstream signaling as a promising therapeutic method for preventing cardiovascular diseases. The findings may advance the current understanding of the effects of AGE on vascular remodeling and contribute to the study of vascular diseases and diabetes.
